# Analysis of four DLX homeobox genes in autistic probands

**DOI:** 10.1186/1471-2156-6-52

**Published:** 2005-11-02

**Authors:** Steven P Hamilton, Jonathan M Woo, Elaine J Carlson, Nöel Ghanem, Marc Ekker, John LR Rubenstein

**Affiliations:** 1 Department of Psychiatry, University of California, San Francisco, CA, USA; 2 Center for Human Genetics, University of California, San Francisco, CA, USA; 3 Genomics Core Facility, University of California, San Francisco, CA, USA; 4 Nina Ireland Laboratory, University of California, San Francisco, CA, USA; 5 Department of Biology, University of Ottawa, Ontario, Canada

## Abstract

**Background:**

Linkage studies in autism have identified susceptibility loci on chromosomes 2q and 7q, regions containing the DLX1/2 and DLX5/6 bigene clusters. The DLX genes encode homeodomain transcription factors that control craniofacial patterning and differentiation and survival of forebrain inhibitory neurons. We investigated the role that sequence variants in DLX genes play in autism by in-depth resequencing of these genes in 161 autism probands from the AGRE collection.

**Results:**

Sequencing of exons, exon/intron boundaries and known enhancers of DLX1, 2, 5 and 6 identified several nonsynonymous variants in DLX2 and DLX5 and a variant in a DLX5/6intragenic enhancer. The nonsynonymous variants were detected in 4 of 95 families from which samples were sequenced. Two of these four SNPs were not observed in 378 undiagnosed samples from North American populations, while the remaining 2 were seen in one sample each.

**Conclusion:**

Segregation of these variants in pedigrees did not generally support a contribution to autism susceptibility by these genes, although functional analyses may provide insight into the biological understanding of these important proteins.

## Background

Autism is a severe heterogeneous neurobehavioral syndrome that becomes apparent in the first years of life [1-3] Autism is often viewed as a type of mental retardation, as most autistics have IQs lower than 70. However, autism is distinguished from other mental retardation syndromes by disproportionately severe deficits in language and social skills. Persons with some autistic features but with preserved language are often referred to as having Asperger's Syndrome [4].

There has been much interest and work investigating the genetic basis of autism [5]. Twin studies have shown that autism is a strongly inherited disorder [2,6,7], as monozygotic twins are concordant for this syndrome substantially more frequently than are dizygotic twins. For reasons that are not yet understood, autism affects boys about four times more often than girls. Non-genetic etiological factors are under careful consideration [8], given the controversy over changing estimates of the incidence of autism over the past decades [9].

Recently, we hypothesized that some forms of autism may be due to a disproportionate high level of excitation (or disproportionately weak inhibition) in neural circuits that mediate language and social behaviors [10]; related models have also been postulated [11,12]. A "noisy" (hyperexcitable, poorly functionally differentiated) cortex is inherently unstable, and susceptible to epilepsy, a common malady of autistic people [13]. Consistent with this model, a subset of patients with a mutation of the X-linked homeobox gene ARX have autistic features [14,15]. Analysis of the expression and function of murine Arx has revealed that this transcription factor controls proliferation of cortical progenitor cells and it regulates the development of the basal ganglia and the tangentially-migrating GABAergic neurons, derived from this region, that become cortical local circuit neurons [16]. Furthermore, Arx is expressed in mature cortical local circuit neurons [16,17]. Arx expression in GABAergic neurons in the basal ganglia and cortex could underlie the epilepsy and movement disorders in humans bearing Arx mutations.

The highly conserved Dlx1, 2, 5 & 6 homeobox transcription factors also control the development of the basal ganglia and cortical local circuit neurons [18-23]. This gene family regulates the expression of glutamic acid decarboxylase [24,25] (and Cobos and Rubenstein, unpublished). Furthermore, these genes control the expression of Arx in cells derived from basal ganglia progenitor domains [17]. Finally, mice lacking Dlx1 have defects in subsets of cortical local circuit neurons that lead to their apoptosis and subsequent onset of epilepsy [26]. Therefore, murine Dlx genes have a central role in controlling the development and function of forebrain GABAergic (inhibitory) neurons. The human DLX genes are organized as bigene clusters on chromosomes 2q31.1 (DLX1/2) and 7q21.3 (DLX5/6) and their expression is controlled by intra- and extragenic enhancers [27-30] These genes are close to loci that have been linked with autism (2q32, and 7q22-31) [31-34].

In a genome scan using 152 affected sibling pairs, a multipoint maximum lod score (MLS) of 3.74 was reported near D2S2188 [31]. This region on chromosome 2 has also been reported to be linked to autism in one study of 51 multiplex families [35], while other studies show linkage slightly more telomeric to this region [36,37]. Chromosome 7q, also an important region in linkage studies of autism [38], contains DLX5 and DLX6. The International Molecular Genetic Study of Autism Consortium (IMGSAC) reported a single locus MLS of 2.93 in 83 sibling pairs at D7S477, some 4.1 Mb telomeric to the DLX5/6 cluster on 7q22 [31]. When 152 sibling pairs were analyzed, a multipoint MLS of 3.20 was calculated. An earlier IMGSAC study reported linkage ~35 Mb telomeric to the DLX5/6 region [39].

Converging biological and genetic evidence thus suggest a role for DLX proteins in autism. While previous studies have failed to identify non-synonymous mutations in DLX1 and DLX2 [32], or close linkage of a polymorphism in DLX6 in autistic patients [40], we sequenced the exons, exon/intron boundaries, and known regulatory elements of the DLX1/2 and DLX5/6 genes in 161 autism probands and 58 non-autistic siblings collected as part of the Autism Genetic Resource Exchange (AGRE) [41], an initiative coordinated by Cure Autism Now (CAN). We identified three non-synonymous variants in DLX2 and two non-synonymous variants in DLX5 that were either very rare or not present in other populations. A number of other SNPs were identified in all four genes.

## Results

We resequenced the coding regions (total 3,153 bp, mean 263 bp/coding region, range 82–400 bp) and flanking non-coding regions (total 3831 bp, mean 319 bp/non-coding region, range 176–755 bp) of human DLX1, DLX2, DLX5, and DLX6. Additionally, we resequenced 2,679 bp of the regions between each bigene cluster known to contain highly conserved tissue specific enhancers [28]. Finally, we sequenced 1,372 bp of an upstream regulatory element (URE2) located ~12 kb upstream of DLX1 (Ghanem and Ekker, unpublished observations).

**Figure 1:**
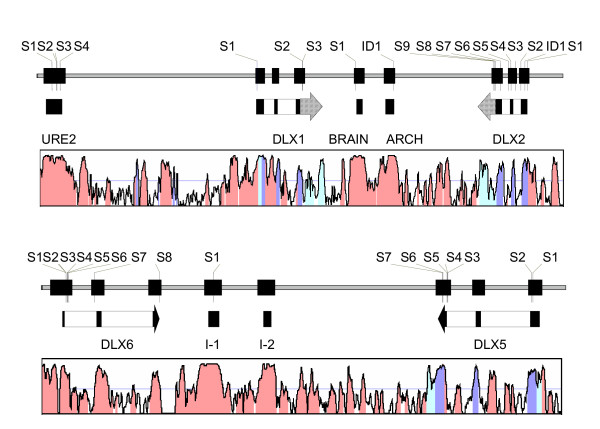
**Schematic of the genomic region for four DLX genes**. The regions depicted are a) chromosome 2q31.1 (33.0 kb) for DLX1 and DLX2; b) chromosome 7q21.3 (20.1 kb) for DLX5 and DLX6. For both regions, the orientation has the telomere to the right of the figure. The top part of each figure shows the position and relative size of the amplicons sequenced, with the resulting SNPs (S) or insertion/deletion (ID) depicted with a thin vertical line. Below this is a schematic of the transcript in it's genomic context, with coding regions in gray and non-coding regions cross-hatched. Finally, a VISTA alignment of the genomic regions is depicted, comparing the human July 2003 build with the mouse February 2003 build, with regions with >75% sequence identity shaded. Exons for DLX6 do not show up as blue, as it was not annotated on the UCSC RefSeq track.

The sample consisted of 161 autism probands and 58 non-autistic siblings obtained from AGRE, for a total of 2.08 Mb of sequence. The DNA variants discovered are summarized in Table 1. The relative location of each variant within the DLX1/2 and DLX5/6 clusters is depicted in Figure 1. Variants are referred to by our simple name (e.g., DLX2 SNP-1) for the purpose of clarity within this manuscript, while technically accurate nomenclature [42,43] or dbSNP rs#, if available, are also referenced in Table 1. Allele frequencies for these variants are detailed in Additional File 2. Thirty-three variants were observed, consisting of 31 single nucleotide polymorphisms (SNPs) and two insertion/deletion polymorphisms. Coding region variants consisted of three synonymous SNPs (one in DLX2, two in DLX5) and four non-synonymous SNPs (two in DLX2, two in DLX5). Additionally, an in-frame insertion of AGC in exon 1 of DLX2 leads to the insertion of an additional serine after serine 46, lengthening a poly-serine stretch from six to seven residues. DLX1 and DLX6 did not have any coding region variants in this study. One SNP each in DLX1 and DLX2, as well as two SNPs in DLX6, have been previously deposited in dbSNP. Interestingly, DNA variants were ~2.3 fold less common in the four DLX1/2 and DLX5/6 intergenic enhancers and DLX1/2 URE2. For 33 of 34 variants, for which corresponding chimpanzee genomic sequence was available, the major human allele matched the chimpanzee allele. For the single exception, in the brain specific DLX1/2 intergenic enhancer, the minor allele seen in our sample matches the allele seen in chimp, mouse, rat, and chicken. We observed none of three SNPs in DLX1 and all three SNPs in DLX2 observed in a recent resequencing effort in 48 autism probands [32].

**Table 1:** DNA variants identified in DLX genes

**Gene**	**Location**	**SNP #**	**Name**	**dbSNP**	**Contig**	**Build34**	**Change**	**AA Pos**	**AA Change**
					NT_005403.14				
DLX1-2									
URE2	-	1	NT_005403.14:g.23146815		23146815	173139942	**C **-> T	-	-
	-	2	NT_005403.14:g.23147084		23147084	173140211	**A **-> G	-	-
	-	3	NT_005403.14:g.23147129		23147129	173140256	**G **-> A	-	-
	-	4	NT_005403.14:g.23147332		23147332	173140459	**G **-> A	-	-
									
DLX1	5'	1	-111G>T		23159712	173152839	**G **-> T	-	-
(AY257976)	3'	2	*170T>C		23162572	173155699	**T **-> C	-	-
	3'	3	*182C>G	rs3821186	23162584	173155711	**C **-> G	-	-
									
DLX1-2 BR	-	1	NT_005403.14:g.23165866		23165866	173158993	C -> **A**	-	-
									
DLX1-2 AR	-	InDel-1	NT_005403.14:g.23168271		23168271	173161398	**G **-> -	-	-
									
DLX2	5'	1	-36G>A	rs743605	23176719	173169846	**G **-> A	-	-
									
(NM_004405)	Exon 1	InDel-1	138_139insAGC		23176546	173169673	AGC	46_47	Ser46_Leu47insSer
	Exon 1	2	394G>A		23176290	173169417	**G **-> A	132	Glu -> Lys
	Intron 1	3	401-156C>T		23175947	173169074	**C **-> T	-	-
	Exon 2	4	525A>G	rs2228184	23175667	173168794	**A **-> G	175	Gln -> Gln
	Intron 2	5	585+100C>G		23175507	173168634	**C **-> G	-	-
	Exon 3	6	670G>A		23175005	173168132	G -> A	224	Ala -> Thr
	3'	7	987*1C>T		23174687	173167814	**C **-> T	-	-
	3	8	987*65C>T		23174623	173167750	**C **-> T	-	-
	3'	9	987*154C>T		23174534	173167661	**C **-> T	-	-
									
					NT_007933.13				
DLX5	Exon 1	1	252C>G		21887602	96265415	**C **-> G	84	Ala -> Ala
(NM_005221)	Exon 1	2	306C>T		21887548	96265361	**C **-> T	102	His -> His
	Intron 2	3	541-44C>G		21884339	96262152	**C **-> G	-	-
	Intron 2	4	541-31C>T		21884326	96262139	**C **-> T	-	-
	Intron 2	5	541-10C>T		21884305	96262118	**C **-> T	-	-
	Exon 3	6	685T>C		21884151	96261964	**T **-> C	229	Ser -> Pro
	Exon 3	7	702C>A		21884134	96261947	**C **-> A	234	Ser -> Arg
									
DLX5-6 I1	-	1	NT_007933.13:g.21875347		21875347	96253160	**A **-> G	-	-
									
DLX6	Intron 1	1	82+57C>T		21869700	96247513	**C **-> T	-	-
(NM_005222)	Intron 1	2	82+58C>T		21869701	96247514	**C **-> T	-	-
	Intron 1	3	82+59C>T		21869702	96247515	**C **-> T	-	-
	Intron 1	4	82+103C>T		21869746	96247559	**C **-> T	-	-
	Intron 1	5	82+124C>T		21869767	96247580	**C **-> T	-	-
	Intron 1	6	82+160C>T		21869803	96247616	**C **-> T	-	-
	Intron 1	7	83-85A>C	rs1207727	21870783	96248596	**A **-> C	-	-
	3'	8	*9A>G	rs3213654	21873286	96251099	**A **-> G	-	-

^a^Variant naming by accepted mutation nomenclature convention (references 53 and 54).

^b^Contig accessions: NT_005403.14 for DLX1/2 region; NT_007933.13 for DLX5/6 region.

^c^Nucleotide changes are listed major allele to minor allele, with chimp allele **bolded**.

^d^Chimp sequence not available for DLX2 InDel-1.

^e^rs2228184 is also known as rs12619503.

We focused on the non-synonymous SNPs and the DLX2 serine insertion for further analysis given the plausibility of the functional significance of these amino acid changes. The DLX2 InDel-1 was observed on 3 autism chromosomes. DLX2 SNPs-2 and 6 and DLX5 SNP-6 were each seen on individual chromosomes, while DLX5 SNP-7 was observed on 4 chromosomes. These observations result in allele frequencies of 0.009, 0.003, 0.003, 0.003, and 0.012, respectively, in our autism sample. We compared the allele frequencies for each of these five observed DLX2 and DLX5 variants in the AGRE sample with the frequency of these DLX variants in a different population. For this purpose we sequenced 188 DNAs from the Coriell DNA Polymorphism Discovery Resource (PDR). This population is not phenotypically defined, but does represent a diverse array of populations for the detection of uncommon DNA polymorphisms. An additional two SNPs (SNPs 3 and 4) in DLX5 were observed in the PDR sample as part of this effort, but not in the autistic sample or the non-autistic siblings (Additional File 2). Additionally, we genotyped the four non-synonymous SNPs in 95 Caucasian and 95 African-American samples, also obtained from the Coriell Institute. No heterozygous genotypes were seen for SNPs 2 and 6 of DLX2. For SNPs 6 and 7 of DLX5, single heterozygous samples were seen, with both occurring in the Caucasian sample. Inspection of this data indicates that allele frequencies for the 4 protein-changing variants did not differ between autistic probands and their siblings, nor between autistic probands and the PDR and human variation panels.

In general, the variants were uncommon. Fifteen were observed only once in the autism probands. Ten were seen in 2–4 autistic individuals. Four of the variants were more common in the autism probands, with minor allele frequencies ranging between 3.4% and 46.5%.

Additional File 3 depicts the genotypes of non-synonymous DLX2 and DLX5 variants and the DLX5/6 intergenic enhancer variant in AGRE pedigrees in which they were detected. The segregation patterns for these variants do not clearly support the hypothesis that they are autism susceptibility variants. DLX2 InDel-1 and DLX2 SNPs-2 and DLX5 SNP-6 are found in affected and unaffected siblings within the same pedigrees. The minor variant for DLX2 SNP-6, which was also seen in the Caucasian panel, is introduced to one of two affected cousins by a father who is not related by blood to the second cousin. SNP-7 in DLX5, which predicts a substitution of Arginine for Serine at position 234, was seen in 3 pedigrees (Additional File 3, figure 1e), but also in the PDR and Caucasian panels. Four autism probands and one sibling diagnosed as broad-spectrum autism were heterozygous for this SNP. Two normal siblings and one autistic proband were homozygous for the wild type allele. It is transmitted to both affected siblings in two families, and to an affected sibling and unaffected sibling in a third. The rich phenotypic data collected by AGRE provided an opportunity to assess any relationship between phenotype and the variants described above. The average non-verbal IQ for all affected persons in the pedigrees in which the four non-synonymous SNPs occurred was 96.5. The two lowest scores, 68 and 85, occurred in two sibs (one with autism, the other with "not quite autism") who both were heterozygous for the DLX2 Serine insertion/deletion and both had early trigonocephaly which normalized. The most notable observation is with family AU0028, in which a heterozygous mother transmits the variant DLX5 SNP-7 to one of her two affected children. Both children have medical histories positive for generalized tonic-clonic seizures, although only the heterozygous child, required medication treatment. Both children also exhibited obsessive-compulsive disroder and attention deficity hyperactivity disorder (ADHD) symptoms, although only the heterozygous child received an ADHD diagnosis. The child heterozygous for the variant also exhibited failure to thrive and an early gait abnormality, which resolved. Given the discordance between genotypes of the affected siblings, it is hard to argue for the role of the DLX5 variant in these specific phenotypes.

We investigated the evolutionary conservation of this particular amino acid change. As shown in Additional File 4, the Serine at position 234 in humans is conserved in two other mammals and one amphibian, and is substituted with Glycine in one bird species. Phylogenetic analysis shows close DLX5 homology among six vertebrate species, particularly among mammals. Similarly, the Serine at position 229 [which is substituted with Proline (SNP-6)] is conserved among all species examined, except for zebrafish, where the residue is Proline.

## Discussion

In the present report, we focused on the DLX bigene clusters given their importance in forebrain development [18,25] and potential neurophysiological processes underlying autism [10]. We sequenced the coding regions and flanking non-coding regions for DLX1, DLX2, DLX5, and DLX6 in 161 autistic probands and 58 non-autistic siblings. We also sequenced four intergenic enhancers (two each between each cluster [28,30]) and an enhancer sequence ~13 kb upstream of DLX1 (Ghanem and Ekker, unpublished observations). In the gene regions, we identified 28 variants, four of which were previously deposited in public SNP databases. We found five variants that are predicted to change or insert an amino acid in the protein in DLX2 and DLX5. Three synonymous SNPs were also found in these genes. Interestingly, no coding sequence variants, synonymous or non-synonymous, were seen in DLX1 or DLX6. The low frequency of the non-synonymous variants preclude meaningful assessment of correlation between variant and disease in the families analyzed. For example, SNP-2 in DLX2 (Glu→Lys), SNP-6 in DLX2 (Ala→Thr), and SNP-6 in DLX5 (Ser→Pro) were each seen in single pedigrees (Additional File 1). In all three cases, one affected offspring in the pedigree has the variant, while either a second affected member does not, or a non-affected sibling also has the variant. The insertion of Serine residue between Serine 36 and Leucine 47 in DLX2 (InDel-1) occurred in three families, but occurred equally as frequently in autistic individuals as in non-autistic siblings, and was seen in 12 of 376 chromosomes in the polymorphism discovery sample. Interestingly, mouse and rat show tri-peptide insertions (Asn-Ser-Ser and Asn-Ser-Asn, respectively) at this site when compared to human and chimp sequence. Finally, a variant predicting a serine to arginine change in DLX5 (SNP-7), was seen in three pedigrees. In two pedigrees, each containing three offspring, two affected individuals were heterozygous for the variant, while a non-autistic sibling was homozygous for the wild type allele. In the third pedigree containing an affected sibling pair, one individual was heterozygous and the other was homozygous for the wild type allele. This variant was seen in one of 374 chromosomes in the polymorphism discovery sample, as well as in one of 380 chromosomes from the human variation panel samples. The non-synonymous changes from SNPs-2 and 6 in DLX2 and SNP-6 in DLX5 were not seen in 376 polymorphism discovery chromosomes or in the human variation panels. Our study design, involving resequencing of DLX family genes in autistic probands, has identified potentially interesting variants within these genes, but cannot provide statistically meaningful inferences about the effect on populations given our ascertainment scheme using non-independent probands from multiplex families and a rather small number of non-affected siblings.

Given the location of DLX1/2 and Dlx5/6 in relation to autism linkage intervals, other groups have examined whether DNA variants in these genes are significantly associated with autism. The IMGSAC consortium conducted a sequence survey of DLX1 and DLX2 in 48 autistic probands [32], which identified three variants in DLX1 that were not detected in our much larger sample and three of the DLX2 variants seen in our sample. One explanation for the non-overlap in findings is the low allele frequency of most variants found in both studies. For example, our SNP-1 in DLX1 was in a region also sequenced by the IMGSAC group, but our allele frequency was 0.2%, making it highly unlikely that it would be detected by assaying 96 chromosomes [32]. The high frequency SNPs-1 and 4 occurred in both samples. Another reason may be the origin of samples. The AGRE samples are predominantly of U.S. origin [41], while the IMGSAC samples are from a variety of geographically diverse countries [39], raising the possibility of population specific variants. This is particularly the case with rare variants, which are less likely to be shared across populations. Finally, differences in coverage of the gene (coding and non-coding regions), depth of coverage (i.e., sample size), or variant detection technology (e.g., direct sequencing in this report, versus variant detection with denaturing high performance liquid chromatography followed by direct sequencing by Bacchelli *et al*.) may explain the discrepancy in variants described. For example, our DLX1 SNPs-2 and 3 lie outside of the region assayed by IMGSAC.

In another recent study in 99 AGRE pedigrees and 308 other pedigrees, two SNPs in DLX2 were investigated [44]. One of these, rs2228184, corresponding to our DLX2 SNP-4, a synonymous coding sequence variant, showed marginal association to autism. This common variant was equally common in autistic probands and their unaffected siblings in our study (Additional File 2). The second SNP in the study by Rabionet et al., which was not associated with autism, occurs outside of the region we sequenced. These published data and our own do not provide support for the possibility that common variation in the DLX loci is associated with autism.

Another study focusing on DLX6 reported the existence of a CAG repeat in exon 1 of DLX6 after assaying 90 Caucasian samples [45]. Although we sequenced the same region, we did not detect this variation. In any case, the uncommon nature of the DLX variants reported here, even in aggregate, are unlikely to provide the basis for any linkage signal in the DLX gene clusters on chromosomes 2 and 7.

A striking observation in our data was the prominent lack of sequence diversity in the five non-coding regions we investigated. In the 4,000 bp encompassing the four intergenic enhancers and DLX1/2 upstream regulatory element, we found only seven variants. However, given the sequence conservation of these functional elements [28], this result is not surprising. Indeed, three of the four intergenic elements are included in 481 genomic segments greater than 200 bp with 100% conservation of human sequence with mouse and rat [46]. In other words, 0.6% of known ultraconserved sequences can be found in the 0.001% of the genome representing the DLX1/2 and DLX5/6 clusters. These deeply conserved sequences that were not exonic were significantly enriched near genes involved with transcriptional regulation, and in particular, those with Homeobox domains (p < 10^-14^) [46].

Of the variants identified, eight occurred in the coding regions of DLX2 and DLX5. The four variants that change the identity of an amino acid are non-conservative modifications: DLX2 SNP-2 (Glutamic acid to Lysine), DLX2 SNP-6 (Alanine to Threonine), DLX5 SNP-6 (Serine to Proline), and DLX5 SNP-7 (Serine to Arginine). In both DLX2 and DLX5, amino acid substitutions lie in conserved regions of the proteins. DLX2 SNP-2 is just N-terminal of the homeodomain in a region conserved among the human DLX2,3,5 subgroup. This DLX2 residue is conserved between human and chimp, dog, rat, and mouse, and fugu, while chicken, African clawed frog, and zebrafish contain the conservative Aspartic acid at the same position. The amino acids changed by DLX2 SNP-6, DLX5 SNP-6 and DLX5 SNP-7 are adjacent to a Proline-rich domain C-terminal to the homeodomain. The amino acids substituted by DLX5 SNP-6 and SNP-7 are invariant in 5 mammal species, chicken (except for the Serine changed to Arginine by SNP-7), and frog. DLX2 InDel-1 leads to the insertion of seventh Serine residue into a six residue polyserine tract within the conserved DLX2,3,5 DllA domain [21,47]. The functional significance of such a change is unknown.

The functional significance of the three synonymous SNPs (DLX2 SNP-4, DLX5 SNP-1 and DLX5 SNP-2) is uncertain, but cannot be summarily dismissed. For example, such "silent" variants can alter binding sites (exonic splice enhancers, ESE) for proteins involved in RNA splicing [48]. Using ESEfinder, a web-based application designed to analyze exonic sequences to identify potential ESEs responsive to the human SR proteins [49], each of these three variants alter the predicted strength or presence of recognition sites for one or more of several highly conserved and structurally related splicing factors termed Serine/Arginine-rich (SR) proteins (data not shown). For instance, the C to T substitution for DLX5 SNP-2 synonymous change abolishes binding sites for two of three SR proteins located in the region surrounding the SNP. The functional significance of this *in silico *observation is unknown, but highlights the potential importance of DNA variation that does not necessarily alter the primary structure or proteins. As is always the case with the analysis of rare variants, until the functionality of these variants is demonstrated, either through statistical differences in allele frequencies at the population level or through direct functional studies, these variants should not be considered disease mutations.

While the identification of variants that generate non-conserved amino acid changes in DLX2 and DLX5 in autistic people suggests that the DLX genes could contribute to autism susceptibility, there are limitations to our study. First the identified DLX variants identified here are rare variants that could be expected to naturally occur across the human population. While this is a possibility given the low likelihood that any random gene is an autism susceptibility locus [50], we believe that biological and genetic linkage data elevate the *a priori *probability that the DLX genes analyzed here may be autism genes. Furthermore, the nature of the amino acid changes suggests that they could alter the function of the DLX proteins, although direct demonstration of this is currently lacking. A second limitation is that the rarity of the DLX variants precludes the possibility that they account for a significant portion of the genetic susceptibility to autism. A further weakness is that our study did not allow the large-scale population-based case control design that would allow a better estimation of the probability that these variants contribute to causing autism. A third limitation involves the use of unaffected siblings in our mutation screen. These siblings were rigorously phenotyped, but not having clinical autism does not rule out the possibility that they have milder traits representing aspects of the autism phenotype. Thus, it is possible that variants shared by affected and "unaffected" siblings may be functionally significant. In terms of co-occurring medical conditions, we found no evidence for such in five families segregating non-synonymous amino acid variants. A fourth limitation is that the population sample we used to compare the allele frequencies of the autism variants with a "normal" population was not optimal. We used the DNA Polymorphism Discovery Resource sample, which is designed to mirror the sequence diversity of the human population. While this sample allowed us to examine a cross section of global genetic diversity, the use of this sample introduces two major limitations for the interpretation of our data. One is the lack of knowledge of phenotypes in the PDR and human variation samples raises the possibility that we may falsely conclude that a variant seen both in autistic probands and the Coriell samples is not involved with autism when in fact the Coriell samples with the variant unbeknownst to us may have autism or a related phenotype. The second involves the relative enrichment of the PDR sample for non-Caucasian samples when compared to our autism sample, which is predominantly Caucasian. This under-sampling of Caucasians (and consequent lack of power to detect rare variants) in the PDR sample may cause us to falsely attribute a rare variant as autism-related, when it may in reality be merely Caucasian-specific. This may indeed be the case for the two DLX5 non-synonymous variants, which were not seen in the PDR sample, but were seen in the Caucasian panel. Thus by including the Caucasian and African-American human variation panels, we have observed that the variants may be Caucasian-specific, albeit at low allele frequencies.

Despite the caveats described above, we suggest that the non-synonymous DLX SNPs could contribute to autism susceptibility for several reasons. Mice lacking DLX1 have epilepsy [26], a common feature in autistic patients. Heterozygosity of transcription factor mutations is well-known to cause human disease [51]. In mice the dosage of the DLX genes is known to be important in controlling the differentiation of forebrain GABAergic neurons and morphogenesis of craniofacial structures, including the middle and inner ear. Indeed, heterozygosity of DLX2 alters morphogenesis of the skull (Depew and Rubenstein, unpublished), although it is uncertain whether heterozygosity of a DLX gene alters brain function. Given recent evidence that the DLX5 locus is partially imprinted in humans [52], heterozygosity for DLX5 alleles could have profound ramifications. In our own pedigrees, we note that two of three pedigrees segregating the DLX5 non-synonymous SNP-7 show maternal transmission, while the single DLX5 SNP-6 pedigree showed paternal transmission (Additional File 3). Increases in *Dlx5 *expression have been found in mice lacking MECP2 (the Rett Syndrome gene), which are associated with alterations in long-range chromatin organization [53]. Therefore, several recent findings are increasing the likelihood that changes in DLX function/expression are involved in neuropsychiatric disorders.

The fact that 4.4% of autistic probands had non-synonymous DLX2 and DLX5 variants (5% when including the DLX5/6 intergenic enhancer variant) could reflect the multifactorial etiology of autism. Finally, perhaps the variants in DLX2, DLX5 and ARX [16], all of which alter the development of forebrain GABAergic neurons [18-25], are providing a clue that an increase in the ratio of excitation/inhibition underlies some forms of autism [10]. Furthermore, it suggests that one should study other genes within genetic pathways that control the ratio of excitation/inhibition in neural circuits that regulate cognition, memory and emotion [10].

## Conclusion

We carried out in depth resequencing of the exons, exon/intron boundaries and known enhancers of the human homeobox genes DLX1, 2, 5 and 6 and identified four nonsynonymous variants in DLX2 and DLX5 in 4% of families tested. We also observed a variant of unknown significance in the highly conserved DLX5/6intragenic enhancer. Without a larger population controlled for phenotype, we cannot assert that these variants are more common in autism. While it is possible that these potentially functional rare variants may alter DLX gene function, our observations do not support a significant contribution to autism susceptibility. More detailed functional analysis or population analysis (e.g., more comprehensive SNP genotyping performed on a massively large trio sample with statistical evidence for genetic association for some of the identified variants) is needed before these variants in the DLX genes can be connected to autism.

## Methods

### Description of sample

Autism sample details: We used 161 autism probands and 58 non-autistic siblings from 95 families in the Autism Genetic Resource Exchange (AGRE) collection [41]. The phenotypic characterization is comprised of the Autism Diagnostic Interview-Revised (ADI-R), a semi-structured clinical instrument for assessing autism [54] based on DSM-IV criteria. In addition, AGRE assigns the phenotypes of Not Quite Autism (NQA) and Broad Spectrum. The former identifies individuals who are no more ≤1 point from meeting autism criteria on any or all of the 3 content domains (i.e., social, communication, and/or behavior); or, individuals who meet criteria on all 3 content domains, but do not meet criteria on the age of onset domain. Broad Spectrum characterizes persons who show patterns of impairment along the spectrum of pervasive developmental disorders, comprising individuals with mild to severe impairment. Details regarding the interview process and consent are available from the AGRE web site . Permission to access the AGRE sample and phenotypic data was obtained. The self-reported ethnicities of the pedigrees are: 68 Caucasian, 16 unknown, 7 mixed parentage, 2 African-American, and 2 Asian-American. In general, samples were chosen in which there were >1 affected person per pedigree. Subjects were chosen blinded to clinical data, including molecular data, beyond the primary phenotype. At the cost of not detecting an unknown number of additional variants, we chose to sequence more than one autism proband from many of the families as a way of more rapidly identifying variants that are segregating with disease in the families. This does not allow direct comparison with the undiagnosed populations described in the following section as one would see in a case-control association study, which was not the design for this study. The correlated genotypes within families would inflate estimates of the allele frequencies of discovered variants if compared to independent controls. Although we do not formally test for differences in allele frequencies, this could be done by randomly choosing a proband from families with more than one proband for any statistical test.

Undiagnosed population sample details: The prevalence of DNA variations detected in the autism sample was assayed in several populations chosen for representation of the major populations of humans. The DNA Polymorphism Discovery Resource (PDR) sample set, obtained from the Coriell Institute, contains samples from United States residents who have ancestors from the major geographic regions of the world: Europe, Africa, the Americas, and Asia. The European-American group includes non-Hispanic whites; the African-American group includes non-Hispanic blacks; the Americas group includes Mexican-Americans and Native Americans; and the Asian-American group includes individuals whose ancestors came from several countries in East and South Asia. We used 188 of the 450 available samples. Additionally, we used 95 Caucasian and 95 African-American samples from the Coriell Human Variation Panels, which do not overlap with the PDR collection.

### Sequence analysis

Sequencing of DLX genes was based on gene structure information provided by GenBank accessions AY257976 (DLX1), NM_004405 (DLX2), NM_005221 (DLX5), and NM_005222 (DLX6). Target sequence for the DLX1/2 and DLX5/6 intergenic enhancers was identified from consensus sequence data [28,30]. Genomic context and intron/exon boundaries were provided by the UCSC (, July 2003, hg16, NCBI Build 34) and ENSEMBL (, February 2004, v19.34b.2) genome browsers.

Sequences were uploaded into VectorNTI 8 (InforMax, Frederick, Maryland) and PCR primers were designed using Primer3 with the human repeat mispriming library [55]. Primers, primer concentrations, and PCR conditions are listed in Additional File 1. All liquid handling was carried out on a TECAN Genesis robot (TECAN-US, Research Triangle Park, NC). PCR reaction volumes were 10 μl, using one of two PCR reagents. The first was Platinum Taq polymerase (Invitrogen, Carlsbad, CA), containing 10 ng DNA template, 50 mM KCl, 20 mM Tris-Hcl (pH 8.4), 200 uM dNTPs, 1.5 mM MgCl2, 1.25 mM Betaine, and 0.25 units Platinum Taq DNA polymerase. The second was with AmpliTaq Gold, containing 10 ng DNA template, and 5 μl of the 2× AmpliTaq Gold Master Mix (Applied Biosystems, Foster City, CA). Primers were added at the concentrations listed in Additional File 1. Reactions were cycled in 96 well GeneAmp 9700 PCR machines (Applied Biosystems). All amplicons could be amplified with one of two protocols. A "short touchdown" protocol involved 5 minutes at 95°C, followed by 10 cycles of 94°C (0:20), 61°C (0:20, decreasing 0.5°C every cycle), and 72°C (0:45), then followed by 35 cycles of 94°C (0:20), 56°C (0:20), and 72°C (0:45), followed by 10 minutes at 72°C. A "long touchdown" procedure involved 5 minutes at 95°C, followed by 14 cycles of 94°C (0:20), 63°C (0:20, decreasing 0.5°C every cycle), and 72°C (0:45), then followed by 35 cycles of 94°C (0:20), 56°C (0:20), and 72°C (0:45), followed by 10 minutes at 72°C. Excess PCR primers and nucleotides were removed by adding a 2 μl solution containing 1 unit each of shrimp alkaline phosphatase and exonuclease I and incubating at 37°C for 1 hour, followed by denaturation of the enzymes by incubation 90°C for 15 minutes. Sequencing was carried out in 5 μl reactions in a 384 well GeneAmp 9700 PCR machine using the BigDye Terminators v3.1 Cycle Sequencing Kit (Applied Biosystems) containing 400 nM sequencing primer, 1 μl PCR template, 0.25 ul dye terminators, and 0.875 μl 5× sequencing buffer. Reactions were cycled by running at 96°C for one minute, followed by 25 cycles of 96°C (0:10), 50°C (0:05), and 60°C (4:00). Unincorporated dye terminators and residual salts were removed by use of the Montáge SEQ_96 _Sequencing Reaction Cleanup Kit (Millipore, Billerica, MA). Samples were electrophoresed on 3700 or 3730xl DNA Analyzer capillary electrophoresis platforms (Applied Biosystems). Bases were called using the ABI KB Basecaller. Sequencher (Gene Codes, Ann Arbor, MI) was used to edit the called bases. All variants will be submitted to dbSNP at the National Center for Biotechnology Information and the AGRE web site upon acceptance of this manuscript for publication. Predicted protein alignments were carried out in the AlignX utility in VectorNTI, with construction of phylogenetic trees using the neighbor joining method [56].

### SNP genotyping

For SNPs 2 and 6 in DLX2 and SNPs 6 and 7 of DLX5, we designed genotyping assays to determine the presence of these SNPs in the Caucasian and African-American Human Variation Panels. For SNPs 2 and 6 in DLX2 and SNP 7 in DLX5, custom 5'-nuclease (TaqMan) assays were designed by and purchased from Applied Biosystems (primers are listed in Additional File 1). Genotyping was performed by cycling a 40-fold dilution of the primer/probe mix with 2× Universal MasterMix and 20 ng of genomic template in a final reaction volume of 5 μl. Samples were incubated at 95°C for 10 minutes, and then were cycled 40 times at 92°C for 15 seconds followed by 60°C for 1 minute. Samples were then brought to room temperature before fluorescence was read on an Applied Biosystems 7900 Sequence Detection System. DLX5 SNP-6 was genotyped using fluorescence polarization detection of template-directed dye terminator incorporation (FP-TDI), an assay based on single base extension [57]. Briefly, the first step involves polymerase chain reactions (PCR) of 5 microliters (μl) containing 500 nM of the forward and reverse primers (Additional File 1), 20 ng genomic DNA template, 200 μM dNTPs (Roche, Indianapolis, IN), 1 M anhydrous betaine (Acros Organics, Geel, Belgium), 50 mM KCl, 20 mM Tris-HCl (pH 8.4), 1.5 mM MgCl_2_, and 0.25 units Platinum Taq DNA polymerase (Invitrogen, Carlsbad, CA). All primers and TDI probes were designed using Primer3 software and were manufactured by Invitrogen (Carlsbad, CA) [55]. Cycling conditions were: 95°C for 5 minutes, followed by a touchdown protocol of 10 cycles 94°C for 20 seconds, then 61°C for 20 seconds (with -0.5°C increments) and 72°C for 45 seconds, followed by 35 cycles of 94°C for 20 seconds, 56°C for 20 seconds, 72°C for 45 seconds, with a subsequent 10 minute incubation at 72°C. Primers and dNTPs were degraded by addition of a 2 μl solution of E. coli exonuclease I and shrimp alkaline phosphatase, with cycling of 37°C for 90 minutes with inactivation at 95°C for 15 minutes. The final step was the addition of a 13 μl solution containing a final concentration of 0.38 μM TDI probe, 2 μl of 10× TDI Reaction Buffer, 0.5 μl of AcycloTerminator Mix (containing R110 and TAMRA labeled AcycloTerminators, corresponding to the polymorphic base), and 0.025 μl of AcycloPol DNA polymerase (PerkinElmer). This mixture was cycled at 95°C for 2 min, followed by 20 cycles of 94°C for 15 sec and 55°C for 30 sec. Genotypes were read on a Victor 2 plate reader (PerkinElmer). Positive controls for both TaqMan and FP-TDI consisted of sequence-verified samples from the initial sequencing survey.

## Authors' contributions

SPH participated in the design of the experiments, carried out the SNP genotyping, data analyses, multi-species sequence alignment, and co-wrote the manuscript. JMW and EJC designed and carried out the DNA sequencing and sequence analysis. NG and ME guided the analysis on the DLX regulatory elements and provided unpublished information their sequence and function. JLRR conceived of the study, participated in its design and coordination and co-wrote the manuscript. All authors read and approved the final manuscript.

## Supplementary Material

Additional file 2 Allele frequencies of variants detected by resequencing in four DLX genes and DLX1/2 intergenic enhancers. The allele frequency of the minor allele is presented for 161 autism probands (autism sample), 58 normal siblings of 58 autism probands (non-autism sample), and 188 samples from the NIGMS/NHGRI Polymorphism Discovery Resource (PDR sample). Selected variants were assayed in the PD sample, as described in the text. Variants from other autism studies are designated in the Variant column, with numbers of samples tested shown in the Autism Sample column. *, Bacchelli et al., 2003. [reference [32] **, Nabi et al., 2003 [reference [40]. The "intron 1 C/T" SNP currently maps to intron 2, and is also known as rs3801290, and was assayed in 221 affected sibling pairs and 210 discordant sibling pairs in 196 AGRE families. †, a variant reported at dbSNP within the region we sequenced, but that was not seen in our dataset. Of note, DLX2 SNP-1, a common SNP (rs743605) was found to differ significantly in allele frequency between the autistic probands and the polymorphism discovery sample (χ^2 ^= 4.43, df = 1, p = 0.04). However, this SNP has also been genotyped as part of the HapMap project in a sample of 30 CEPH trios, and the minor allele frequency was found to be 0.47, essentially identical to our autism sample. This discrepancy is likely due to the heterogeneous population composition of the polymorphism discovery sample, which is approximately 26% each of European-American, African-American, and Asian-American populations, with smaller contributions of Mexican-American and Native-American samples (Collins, et al., 1998, Genome Res. 8, 1229–1231). Click here for file

Additional file 3 1a AGRE pedigrees segregating InDel-1 DLX2. -, no insertion. AGC, insertion of AGC. ■, autism. ◒, NQA (not quite autism). 1b AGRE pedigrees segregating SNP-2 DLX2. ■, autism. 1c AGRE pedigrees segregating SNP-6 DLX2. ■, autism. 1d AGRE pedigrees segregating SNP-6, DLX5. ■, autism. 1e AGRE pedigrees segregating SNP-7 in the third exon of DLX5. ■, autism; ◓, broad spectrum autism. 1f AGRE pedigrees segregating SNP-1 from the DLX5/6 intergenic enhancer. ■, autism. Click here for file

Additional file 4 Alignment of DLX5 protein sequences in six vertebrates. a) The region corresponding to amino acid residues 219–244 in the human sequence is depicted. Sequences are listed by GenBank accession and species (HS, Homo sapiens; MM, Mus musculus; RN, Rattus norvegicus; GG, Gallus gallus; XL, Xenopus laevis; and DR, Danio rerio). Type color depicts alignment status (red on yellow, completely conserved; blue on cyan, consensus derived from block of similar residues; green, residue weakly similar to consensus residue; black, non-similar to consensus residue; black on green, consensus derived from majority residue). The location of the residue affected by DLX5 SNPs is shown by the arrows (SNP-6, Ser/Pro; SNP-7, Ser/Arg). b) Phlyogenetic tree for the entire DLX5 protein sequence of six vertebrates calculated using neighbor joining method (reference 51, Saitou and Nei, 1987). The distances in parentheses represent the degree of divergence between sequences. Click here for file

Additional file 1 Sequencing and genotyping conditions. PCR primers and conditions for generating templates for DNA sequencing for four DLX genes, two intergenic DLX1/2 enhancers (DLX1-2 BR and DLX1-2 AR), two DLX5/6 intergenic enhancers (DLX5/6 I-1 and I-2), and DLX1/2 upstream regulatory element (URE2). PCR protocols are short touchdown (ST) or long touchdown (LT), as described in Methods. The DNA polymerases (Pol) were Platinum Taq (P) or AmpliTaq Gold (AT). For SNP genotyping, primers for TaqMan (TM) or FP-TDI (FP) are shown. PCR primers and hybridization probes are shown for Taqman, with fluors (VIC or FAM) indicated along with **bolded **polymorphic base. Note inverted nature of DLX5 SNP-6. For DLX5 SNP-6, FP-TDI single base extension probe is shown. Note amplicon for this SNP is same as sequencing amplicon for DLX5 exon 3. Conditions for SNP genotyping are detailed in Methods. Click here for file
