# Supplementary material for: Analysis of four DLX homeobox genes in autistic probands
Source: BMC Genet. 2005 Nov 2;6:52. doi: 10.1186/1471-2156-6-52 (PMC1310613; doi:10.1186/1471-2156-6-52)
Supplement: Additional file 2 — Allele frequencies of variants detected by resequencing in four DLX genes and DLX1/2 intergenic enhancers. The allele frequency of the minor allele is presented for 161 autism probands (autism sample), 58 normal siblings of 58 autism probands (non-autism sample), and 188 samples from the NIGMS/NHGRI Polymorphism Discovery Resource (PDR sample). Selected variants were assayed in the PD sample, as described in the text. Variants from other autism studies are designated in the Variant column, with numbers of samples tested shown in the Autism Sample column. *, Bacchelli et al., 2003. [reference [32] **, Nabi et al., 2003 [reference [40]. The "intron 1 C/T" SNP currently maps to intron 2, and is also known as rs3801290, and was assayed in 221 affected sibling pairs and 210 discordant sibling pairs in 196 AGRE families. †, a variant reported at dbSNP within the region we sequenced, but that was not seen in our dataset. Of note, DLX2 SNP-1, a common SNP (rs743605) was found to differ significantly in allele frequency between the autistic probands and the polymorphism discovery sample (χ2 = 4.43, df = 1, p = 0.04). However, this SNP has also been genotyped as part of the HapMap project in a sample of 30 CEPH trios, and the minor allele frequency was found to be 0.47, essentially identical to our autism sample. This discrepancy is likely due to the heterogeneous population composition of the polymorphism discovery sample, which is approximately 26% each of European-American, African-American, and Asian-American populations, with smaller contributions of Mexican-American and Native-American samples (Collins, et al., 1998, Genome Res. 8, 1229–1231). [file 1471-2156-6-52-S2.pdf]

Gene	Variant	Name	Change	Autism Sample n	Autism Sample freq	Non-Autism Sample n	Non-Autism Sample freq	PD Sample n	PD Sample freq
DLX1-2 URE2	1	NT_005403.14:g.23146815	C -> T	161	0.003	54	0.000	-	-
	2	NT_005403.14:g.23147084	A -> G	161	0.006	58	0.000	-	-
	3	NT_005403.14:g.23147129	G -> A	162	0.006	58	0.017	-	-
	4	NT_005403.14:g.23147332	G -> A	162	0.003	58	0.009	-	-
DLX1	1	-111G>T	G -> T	161	0.000	54	0.009	-	-
	*	"142-284"	G -> A	48	0.010				
	*	"142-266"	T -> A	48	0.010				
	2	*170T>C	T -> C	160	0.003	54	0.000	-	-
	3	*182C>G	C -> G	160	0.006	54	0.009	-	-
DLX1-2 BR	1	NT_005403.14:g.23165866	C -> A	157	0.207	48	0.219	-	-
DLX1-2 AR	InDel-1	NT_005403.14:g.23168271	G -> -	161	0.003	54	0.000	-	-
DLX2	1	-36G>A	G -> A	158	0.465	54	0.481	188	0.383
	†	rs6147027	48bp InDel						
	InDel-1	138_139insAGC	- -> AGC	158	0.009	54	0.019	188	0.032
	†	rs4410254	C -> A						
	2	394G>A	G -> A	157	0.003	52	0.010	188	0.000
	3	401-156C>T	C -> T	161	0.009	54	0.000	-	-
	4	525A>G	A -> G	161	0.410	54	0.398	-	-
	†	rs12616215	T -> C						
	5	585+100C>G	C -> G	53	0.000	54	0.009	-	-
	†	rs12616187	T -> C						
	6	670G>A	G -> A	161	0.003	53	0.000	188	0.000
	7	987*1C>T	C -> T	161	0.006	53	0.019	188	0.005
	8	987*65C>T	C -> T	161	0.003	53	0.009	188	0.000
	9	987*154C>T	C -> T	161	0.003	53	0.009	188	0.000
DLX5	1	252C>G	C -> G	152	0.003	48	0.010	-	-
	2	306C>T	C -> T	152	0.003	48	0.010	-	-
	†	rs1207733	C -> T						
	3	541-44C>G	C -> G	161	0.000	54	0.000	187	0.003
	4	541-31C>T	C -> T	161	0.000	54	0.000	187	0.003
	5	541-10C>T	C -> T	161	0.003	54	0.000	187	0.003
	6	685T>C	T -> C	161	0.003	54	0.000	187	0.000
	7	702C>A	C -> A	161	0.012	54	0.000	187	0.003
DLX5-6 I1	1	NT_007933.13:g.21875347	A -> G	158	0.003	54	0.000	-	-
DLX6	†	rs5885995	GCA InDel						
	1	82+57C>T	C -> T	150	0.007	53	0.000	-	-
	2	82+58C>T	C -> T	150	0.007	53	0.009	-	-
	3	82+59C>T	C -> T	150	0.003	53	0.000	-	-
	4	82+103C>T	C -> T	150	0.000	53	0.019	-	-
	5	82+124C>T	C -> T	150	0.000	53	0.009	-	-
	6	82+160C>T	C -> T	150	0.003	53	0.000	-	-
	†	rs4141284	T -> G						
	7	83-85A>C	A -> C	160	0.034	53	0.047	-	-
	**	"intron 1 C/T"	C -> T		0.32				
	8	*9A>G	A -> G	161	0.009	51	0.000	-	-
