# Supplementary figures and images for: Analysis of four DLX homeobox genes in autistic probands
Source: BMC Genet. 2005 Nov 2;6:52. doi: 10.1186/1471-2156-6-52 (PMC1310613; doi:10.1186/1471-2156-6-52)

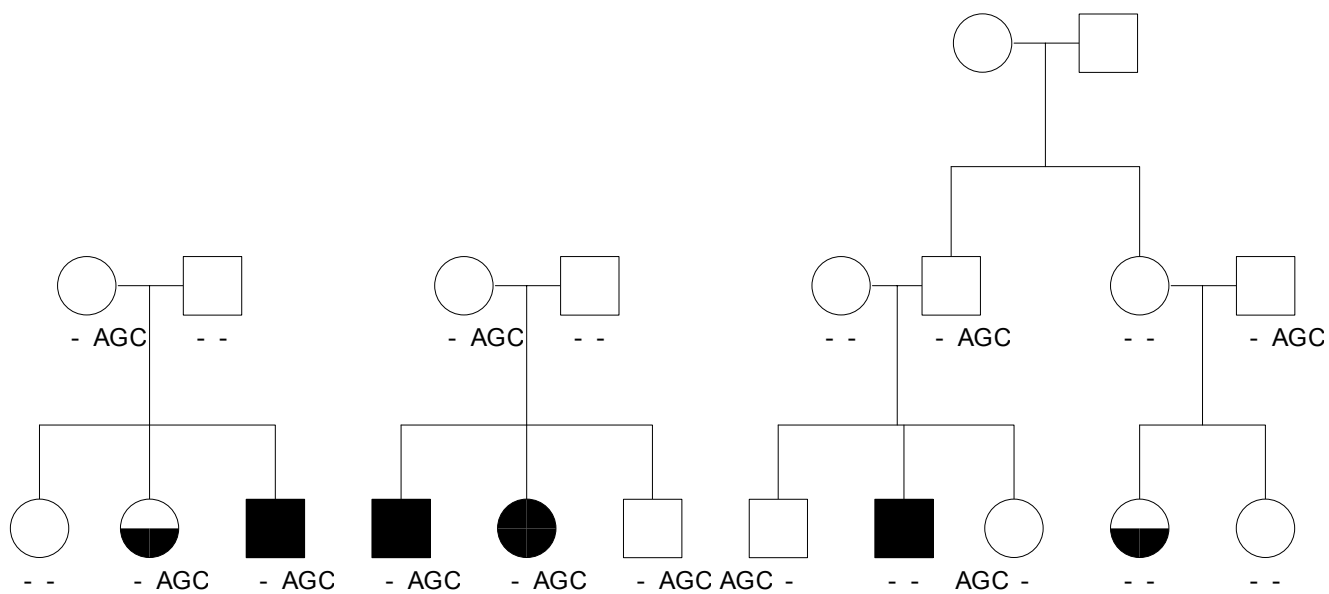

AU0063

AU0084

AU0752

1a

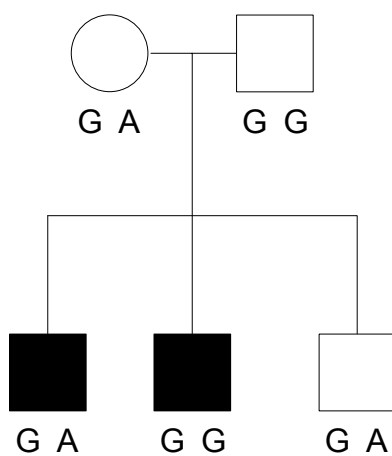

AU0501

1b

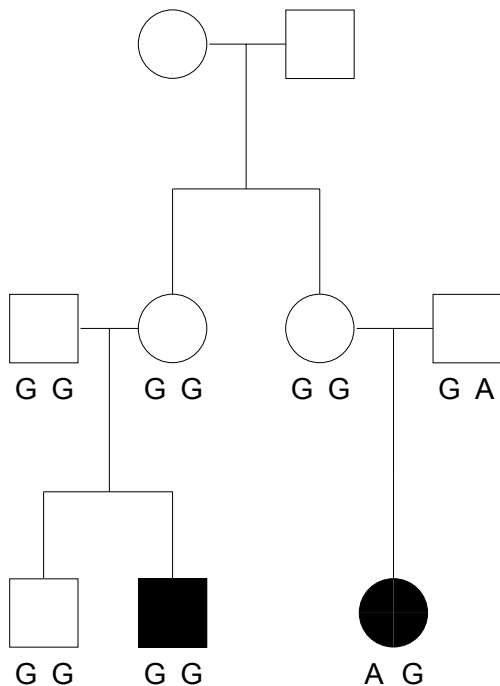

AU0672

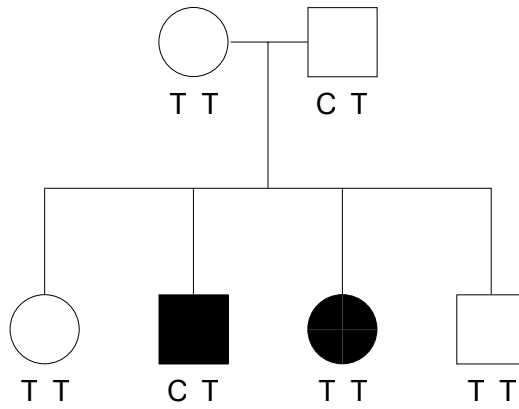

AU0469

1d

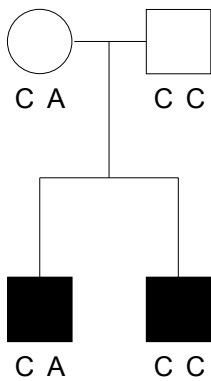

AU0028

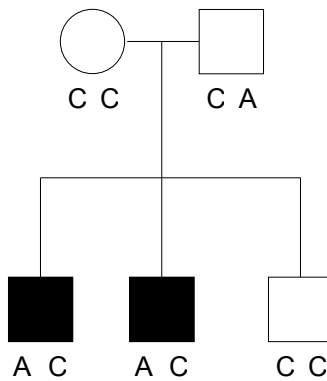

AU0200

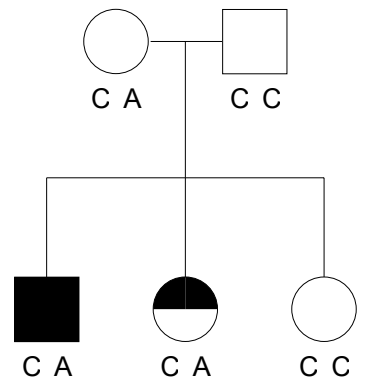

AU0262

1e

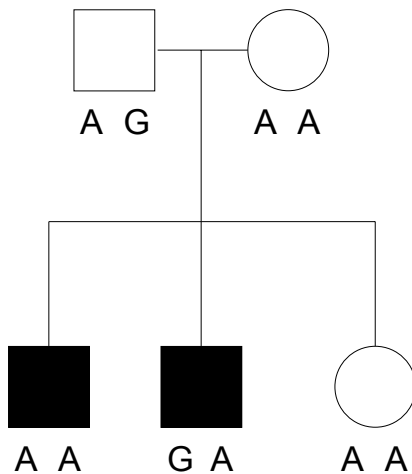

AU0423

Supplement: Additional file 3 — 1a AGRE pedigrees segregating InDel-1 DLX2. -, no insertion. AGC, insertion of AGC. ■, autism. ◒, NQA (not quite autism). 1b AGRE pedigrees segregating SNP-2 DLX2. ■, autism. 1c AGRE pedigrees segregating SNP-6 DLX2. ■, autism. 1d AGRE pedigrees segregating SNP-6, DLX5. ■, autism. 1e AGRE pedigrees segregating SNP-7 in the third exon of DLX5. ■, autism; ◓, broad spectrum autism. 1f AGRE pedigrees segregating SNP-1 from the DLX5/6 intergenic enhancer. ■, autism. [file 1471-2156-6-52-S3.pdf]

a)

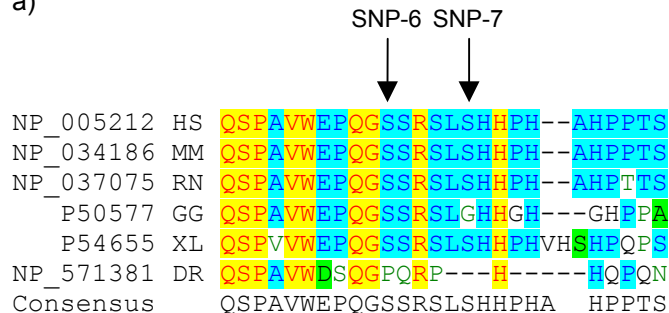

b)

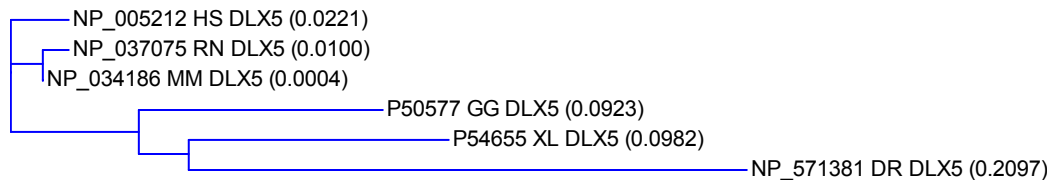

Supplement: Additional file 4 — Alignment of DLX5 protein sequences in six vertebrates. a) The region corresponding to amino acid residues 219–244 in the human sequence is depicted. Sequences are listed by GenBank accession and species (HS, Homo sapiens; MM, Mus musculus; RN, Rattus norvegicus; GG, Gallus gallus; XL, Xenopus laevis; and DR, Danio rerio). Type color depicts alignment status (red on yellow, completely conserved; blue on cyan, consensus derived from block of similar residues; green, residue weakly similar to consensus residue; black, non-similar to consensus residue; black on green, consensus derived from majority residue). The location of the residue affected by DLX5 SNPs is shown by the arrows (SNP-6, Ser/Pro; SNP-7, Ser/Arg). b) Phlyogenetic tree for the entire DLX5 protein sequence of six vertebrates calculated using neighbor joining method (reference 51, Saitou and Nei, 1987). The distances in parentheses represent the degree of divergence between sequences. [file 1471-2156-6-52-S4.pdf]
