# Supplementary material for: Analysis of four DLX homeobox genes in autistic probands
Source: BMC Genet. 2005 Nov 2;6:52. doi: 10.1186/1471-2156-6-52 (PMC1310613; doi:10.1186/1471-2156-6-52)
Supplement: Additional file 1 — Sequencing and genotyping conditions. PCR primers and conditions for generating templates for DNA sequencing for four DLX genes, two intergenic DLX1/2 enhancers (DLX1-2 BR and DLX1-2 AR), two DLX5/6 intergenic enhancers (DLX5/6 I-1 and I-2), and DLX1/2 upstream regulatory element (URE2). PCR protocols are short touchdown (ST) or long touchdown (LT), as described in Methods. The DNA polymerases (Pol) were Platinum Taq (P) or AmpliTaq Gold (AT). For SNP genotyping, primers for TaqMan (TM) or FP-TDI (FP) are shown. PCR primers and hybridization probes are shown for Taqman, with fluors (VIC or FAM) indicated along with bolded polymorphic base. Note inverted nature of DLX5 SNP-6. For DLX5 SNP-6, FP-TDI single base extension probe is shown. Note amplicon for this SNP is same as sequencing amplicon for DLX5 exon 3. Conditions for SNP genotyping are detailed in Methods. [file 1471-2156-6-52-S1.pdf]

Gene	Location	Coding bp	Non-coding bp	bp	Forward	Reverse	primer conc. (μM)	PCR prg.	Pol
	**DNA Sequencing**								
DLX1-2 URE2-a	N/A	0	1372	1372[a]	AATCAGACCCCTACTGGGAAAA	CACGCCTGTTCCTCTGTGTAG	1	ST	AT
DLX1-2 URE2-b	N/A				CTTTGTAGCAAGTGCAACTCCA	TGGGACTGACTGATCTGGTTTT	2	ST	AT
DLX1	Exon 1	313	274	587	TTTATGACCTTCGCTGAGTCAA	TTTCCCTCTCTCCCTTGTACC	2	ST	P
	Exon 2	200	224	424	CTTCCCTGGCTTTCAGAGTTT	CAGCGAGCAACAAATGAAGA	5	ST	AT
	Exon 3	255	427	682	GGTTGGGAGGTCCTATCTCTG	CCTGATAGTCCCACAAAGCTG	5	LT	AT
DLX1-2 BR	N/A	0	656	656	AGTCTGTAATGGCAAAATGCAA	AAAGCCAAGACCAGTGAGGA	5	ST	AT
DLX1-2 AR	N/A	0	685	685	CCAGACCCATCCTCCTATCTT	GGCCAATGCTCTATTTGAGG	2	ST	AT
DLX2	Exon 1	400	234	634	CTTTCTTGTTTCCCCACCAC	AGCGTGAAAATCCAGAGCTT	5	ST	P
	Exon 2	185	359	544	TTGCCACCAGCTTCTTTCTC	CAGGCCTGGAAATCCTTAAAA	5	ST	AT
	Exon 3	402	290	692	CCTACTGCCCCAAGACTAGGT	GTCAGAAATGCGGCCTTTTA	5	ST	P
DLX5	Exon 1	355	176	531	CCACAGCCATGTCTGCTTAG	CAATCTACCACCCCATCTCG	1.25	ST	AT
	Exon 2	185	297	482	CATATTTATCGCGGTCCTGAA	AGCATAGGGGCTGATGTCTTT	0.625	ST	AT
	Exon 3	330	243	573	GCATAGCTTCTTGGCGGTAG	CTTCCCCATATGAATTCCTTT	5	ST	P
DLX5-6 I-1	N/A	0	671	671	CATACAGTCGTGCAAAAGGACA	TGTTGGTTTCGATGTATGGTAGC	2	ST	P
DLX5-6 I-2	N/A	0	667	667	AAAGCAAAGTTGTAGCCCATGA	TCTTTTGTCCTTCAGGGTTTGA	4	ST	P
DLX6	Exon 1	82	755	837	TGCAAGGATCCAAAGAGCTAAG	GAGAGCGCAGTGTGGACTAACT	4	ST	P
	Exon 2	194	304	498	AGGGACTGGGTCAAGGAGAT	CTTCCATCCTTGGTTGACTAGG	0.625	ST	AT
	Exon 3	252	248	500	GAGGCTAGAAAGAGGTGGAAGG	CCTTTTCGCAGATACAAGCAG	0.625	ST	AT
	**SNP Genotyping**								
DLX2 SNP-2	Exon 1	TM	PCR	70	CGCTCCCTATGGAACCAGTTC	ACTCTTGACTTCAGCGTTATGCA			
	G>A	TM	Probes		VIC-CCAACAAC**G**AGCCTG	FAM-CAACAAC**A**AGCCTG			
DLX2 SNP-6	Exon 3	TM	PCR	79	AGAAGATGTGGAAAAGTGGTGAGATC	GGCGGCGAAGCACAAG			
	G>A	TM	Probes		VIC-AAGCGCTGG**C**CCCA	FAM-AAGCGCTGG**T**CCCA			
DLX5 SNP-6	Exon 3	FP	PCR	573	GCATAGCTTCTTGGCGGTAG	CTTCCCCATATGAATTCCTTT			
	T>C	FP	Probe		GTGTGGGAGCCCCAGGGC				
DLX5 SNP-7	Exon 3	TM	PCR	69	TCTCCAGCGGTGTGGGA	CGGAGGGTGGGCATGAG			
	C>A	TM	Probes		VIC-CTCGCTCAG**C**CACCAC	FAM- CGCTCAG**A**CACCAC			

[a] Overlapping amplicons cover a total of 1372 base pairs.
